# Supplementary material for: Insecticidal and Detoxification Enzyme Inhibition Activities of Essential Oils for the Control of Pulse Beetle, Callosobruchus maculatus (F.) and Callosobruchus chinensis (L.) (Coleoptera: Bruchidae)
Source: Molecules. 2023 Jan 4;28(2):492. doi: 10.3390/molecules28020492 (PMC9863611; doi:10.3390/molecules28020492)
Supplement: Supplementary file 1 [file molecules-28-00492-s001.zip › molecules-2074531-supplementary.pdf]

Supplementary material

Insecticidal and detoxification enzyme inhibition activities of essential oils for the control of pulse beetle, *Callosobruchus maculatus* (F.) and *Callosobruchus chinensis* (L.) (Coleoptera: Bruchidae)

Himanshi Gupta [1], Deeksha [1], Urvashi [1,2], S. G. Eswara Reddy [1,2*]

[1] Entomology Laboratory, Agro-technology Division, CSIR-Institute of Himalayan Bioresource Technology, Palampur 176061, India
[2] Academy of Scientific and Innovative Research (AcSIR), Ghaziabad 201002, India
* Correspondence: ereddy@ihbt.res.in

Table S1. Chemical composition of essential oil of *H. spicatum.*

Sr. No.	Name	RI[a]	RI[b]	Area (%)	Mode of Identification
1	β–pinene	974	982	3.10	MS, RI
2	α–phellandrene	1002	989	2.80	MS, RI
3	1,8–cineole	1026	1039	28.31	MS, RI
4	Linalool	1095	1103	0.50	MS, RI
5	α–copaene	1374	1361	0.92	MS, RI
6	β–elemene	1389	1390	0.46	MS, RI
7	*trans*–β–cryophyllene	1408	1413	0.58	MS, RI
8	α–gurjunene	1409	1424	2.23	MS, RI
9	*trans*–β–farnesene	1440	1433	0.15	MS, RI
10	α–humulene	1452	1452	2.01	MS, RI
11	γ–murrolene	1478	1479	3.87	MS, RI
12	α–murrolene	1500	1504	2.93	MS, RI
13	δ–cadinene	1527	1521	11.09	MS, RI
14	α–calacorene	1544	1543	0.51	MS, RI
15	Elemol	1548	1550	0.74	MS, RI
16	*cis*–nerolidol	1561	1560	4.59	MS, RI
17	Germacrene–D–4–ol	1574	1565	0.89	MS, RI
18	Caryophyllene oxide	1582	1587	0.60	MS, RI
19	Viridiflorol	1592	1594	1.51	MS, RI
20	10–epi–γ–eudesmol	1622	1622	2.45	MS, RI
21	1–epi–cubenol	1629	1627	1.37	MS, RI
22	γ–eudesmol	1630	1639	5.74	MS, RI

23	α–eudesmol	1652	1658	18.83	MS, RI
	Unidentified	–	–	2.82	
	Total			96.18	
	Monoterpene hydrocarbons*			34.21	
	Oxygenated monoterpene*			0.50	
	Sesquiterpene hydrocarbons*			24.75	
	Oxygenated sesquiterpene*			36.72	

[a] Retention index value of compounds in the literature
[b] Retention index value determined relative to n–alkanes (C9–C24) on the DB–5 GC column
* Percentage of compounds class in analyzed essential oil samples

Table S2. Chemical composition of essential oil of *C. deodara.*

Sr. No.	Name	RI[a]	RI[b]	Area (%)	Mode of Identification
1	Longifalene	1414	1414	0.63	MS, RI
2	α–himachalene	1449	1453	18.11	MS, RI
3	γ–murrolene	1478	1478	0.48	MS, RI
4	α–dehydro–ar–himachalene	1516	1519	44.81	MS, RI
5	β–selinene	1489	1493	2.39	MS, RI
6	Longiborneol	1599	1591	0.20	MS, RI
7	β–himachalene oxide	1625	1615	0.17	MS, RI
8	*Trans*–β–elemenone	1602	1600	0.15	MS, RI
9	γ–curcumene	1481	1490	9.33	MS, RI
10	α–acorenol	1632	1633	0.36	MS, RI
11	Z–γ–atlantone	1694	1697	4.81	MS, RI
12	α–Z–atlantone	1717	1710	8.09	MS, RI
	Total			89.53	
	Monoterpene hydrocarbons*			–	
	Oxygenated monoterpene*			–	
	Sesquiterpene hydrocarbons*			76.55	
	Oxygenated sesquiterpene*			12.9	

[a] Retention index value of compounds in the literature
[b] Retention index value determined relative to n–alkanes (C9–C24) on the DB–5 GC column
* Percentage of compounds class in analyzed essential oil samples

Table S3. Chemical composition of essential oil of *J. communis.*

Sr. No.	Name	RI[a]	RI[b]	Area (%)	Mode of Identification
1	Camphene	956	941	61.03	MS, RI
2	Sabinene	969	974	0.33	MS, RI
3	β–pinene	974	981	0.79	MS, RI
4	Myrcene	988	990	3.09	MS, RI
5	δ–3–carene	1012	1012	0.23	MS, RI
6	Dl–limonene	1024	1036	27.91	MS, RI
7	α–terpinolene	1086	1086	0.37	MS, RI
8	Cedrene	1410	1421	0.74	MS, RI
9	Caryophyllene	1417	1423	0.20	MS, RI
10	β–cadrene	1419	1429	0.24	MS, RI
11	Widdrene	1429	1439	0.17	MS, RI
12	α–himachalene	1449	1451	0.08	MS, RI
13	α–humulene	1452	1460	0.12	MS, RI
14	γ–himachalene	1481	1468	0.05	MS, RI
15	Germacrene–D	1480	1470	0.06	MS, RI
16	Himachalene β	1500	1485	0.25	MS, RI
17	δ–cadinene	1522	1522	0.26	MS, RI
18	Germacrene–B	1559	1567	0.18	MS, RI
19	Cedrol	1618	1624	1.85	MS, RI
20	Abietadiene	2097	2099	0.15	MS, RI
	Total			96.25	
	Monoterpene hydrocarbons*			93.75	
	Oxygenated monoterpene*			–	
	Sesquiterpene hydrocarbons*			2.5	
	Oxygenated sesquiterpene*			–	

[a] Retention index value of compounds in the literature

[b] Retention index value determined relative to n–alkanes (C9–C24) on the DB–5 GC column

* Percentage of compounds class in analyzed essential oil samples

Table S4. Chemical composition of essential oil of *J. recurva.*

Sr. No.	Name	RI[a]	RI[b]	Area (%)	Mode of Identification
1	α−thujene	924	927	1.71	MS, RI
2	3−octen−5−yne	958	957	13.81	MS, RI
3	2−β−pinene	974	980	39.18	MS, RI
4	β−myrcene	988	990	4.53	MS, RI
5	α−terpinene	1017	1019	1.37	MS, RI
6	Cymene	1020	1027	1.65	MS, RI
7	Dl−limonene	1024	1031	1.55	MS, RI
8	γ−terpinene	1054	1060	2.36	MS, RI
9	α−terpinolene	1087	1086	0.86	MS, RI
10	Terpinen−4−ol	1174	1185	1.42	MS, RI
11	Caryophyllene	1417	1423	0.39	MS, RI
12	δ−cadinene	1522	1521	12.93	MS, RI
13	γ−murrolene	1478	1479	0.96	MS, RI
14	Germacrene−D	1480	1486	0.41	MS, RI
15	Trans−murrola−4(14),5 diene	1493	1497	0.64	MS, RI
16	α−murrolene	1500	1504	2.72	MS, RI
17	γ−cadinene	1513	1521	3.22	MS, RI
18	α−cadinene	1537	1539	0.40	MS, RI
19	Elemol	1548	1544	1.00	MS, RI
20	γ−eudesmol	1630	1638	0.22	MS, RI
21	α−cadinol	1652	1657	1.13	MS, RI
	Unidentified	−	−	7.54	
	Total			99.46	
	Monoterpene hydrocarbons*			54.63	
	Oxygenated monoterpene*			−	
	Sesquiterpene hydrocarbons*			21.67	
	Oxygenated sesquiterpene*			2.35	

[a] Retention index value of compounds in the literature

[b] Retention index value determined relative to n−alkanes (C9−C24) on the DB−5 GC column

* Percentage of compounds class in analyzed essential oil samples

Table S5. Chemical composition of essential oil of *L. angustifolia.*

Sr. No.	Name	RI[a]	RI[b]	Area (%)	Mode of Identification
1	Camphene	946	952	0.44	MS, RI
2	β–myrcene	988	989	2.18	MS, RI
3	dl–limonene	1024	1031	1.33	MS, RI
4	*trans*–β–ocimene	1044	1045	2.01	MS, RI
5	Linalool	1095	1108	33.30	MS, RI
6	Camphor	1141	1452	0.13	MS, RI
7	Lavandulol	1165	1168	0.66	MS, RI
8	Borneol	1165	1179	0.36	MS, RI
9	Terpinen–4–ol	1174	1185	0.45	MS, RI
10	α–terpineol	1186	1201	1.60	MS, RI
11	Geraniol	1249	1254	0.30	MS, RI
12	Linalyl isobutyrate	1373	1375	40.16	MS, RI
13	Geranyl acetate	1379	1378	0.54	MS, RI
14	Trans–cryophyllene	1408	1421	8.41	MS, RI
15	α–trans–bergamotene	1434	1433	0.43	MS, RI
16	*Cis*–β–farnesene	1440	1451	1.50	MS, RI
17	α–humulene	1454	1460	0.34	MS, RI
18	Germacrene–D	1480	1486	0.48	
19	γ–cadinene	1513	1518	0.30	
20	Caryophyllene oxide	1582	1593	0.70	
	Total			95.62	
	Monoterpene hydrocarbons*			8.53	
	Oxygenated monoterpene*			75.36	
	Sesquiterpene hydrocarbons*			11.03	
	Oxygenated sesquiterpene*			0.70	

[a] Retention index value of compounds in the literature
[b] Retention index value determined relative to n–alkanes (C9–C24) on the DB–5 GC column
* Percentage of compounds class in analyzed essential oil samples

Table S6. Chemical composition of essential oil of *P. wallichiana.*

Sr. No.	Name	RI[a]	RI[b]	Area (%)	Mode of Identification
1	Camphene	946	941	46.21	MS, RI
2	Thuja–2,4 (10) diene	953	953	1.19	MS, RI
3	2–β–pinene	974	976	31.78	MS, RI
4	δ –3–carene	1008	1009	0.29	MS, RI
5	α–terpinene	1014	1012	0.48	MS, RI
6	p–cymene	1020	1019	0.64	MS, RI
7	dl–limonene	1024	1033	8.50	MS, RI
8	Linalool	1095	1096	0.18	MS, RI
9	*Trans*–pinocarveol	1135	1147	0.21	MS, RI
10	*Trans*–pinocamphone	1158	1152	0.04	MS, RI
11	Borneol	1165	1165	0.06	MS, RI
12	Terpinen–4–ol	1174	1167	0.05	MS, RI
13	α–terpineol	1186	1185	0.16	MS, RI
14	Linalool acetate	1254	1252	0.35	MS, RI
15	Bornyl acetate	1285	1285	0.15	MS, RI
16	α–longipinene	1350	1353	0.66	MS, RI
17	β–bourbonene	1387	1377	0.05	MS, RI
18	Longifalene	1407	1415	2.12	MS, RI
19	*Trans*–caryophyllene	1408	1423	0.89	MS, RI
20	α–humulene	1452	1460	0.13	MS, RI
21	β–bisabolene	1505	1509	0.05	MS, RI
22	Caryophyllene oxide	1582	1593	0.11	MS, RI
	Total			94.3	
	Monoterpene hydrocarbons*			88.34	
	Oxygenated monoterpene*			1.99	
	Sesquiterpene hydrocarbons*			3.8	
	Oxygenated sesquiterpene*			0.21	

[a] Retention index value of compounds in the literature

[b] Retention index value determined relative to n–alkanes (C9–C24) on the DB–5 GC column

* Percentage of compounds class in analyzed essential oil samples

Table S7. Ovipositional inhibition effect of different oils against adults of *C. maculatus*.

Percent OI (Mean±SD) of *C. maculatus* at different concentrations (µl/L) after 24 h						
Essential oils	10000	5000	2500	1250	625	Pooled mean
A. calamus	32.00±8.80	25.40±8.65	21.00±6.44	20.40±7.47	16.00±7.52	22.96±9.03[bc]
L. angustifolia	37.20±6.06	23.00±6.74	23.00±8.45	20.00±11.79	15.00±7.65	23.64±10.74[bc]
H. spicatum	46.40±10.62	28.80±8.87	25.60±9.23	22.00±7.78	25.80±4.97	29.72±11.73[ab]
C. deodara	30.20±10.06	33.20±4.82	27.60±4.88	22.20±2.59	17.60±3.78	26.16±7.80[b]
P. wallichiana	22.00±12.41	7.80±8.93	15.80±10.80	28.60±5.55	13.40±11.19	17.52±11.75[c]
J. communis	51.20±7.56	41.60±8.90	31.60±7.23	23.40±6.11	20.20±10.92	33.60±13.98[a]
J. recurve	38.80±9.68	34.80±8.70	27.60±12.44	20.20±5.54	10.00±7.52	26.28±13.40[b]
Pooled mean	36.83±12.69[a]	27.80±12.51[b]	24.60±9.37[b]	22.40±7.10[b]	16.70±8.73[c]	25.70±12.16
Oils	$F_{6,174}$=9.38; p<0.0001					
Concentrations	$F_{4,174}$=27.30; p<0.0001					
Oils × Concentrations	$F_{24,174}$=2.54; p<0.0001					

Mean of five replications: Means followed by the same letter within a column are not statistically significant (p>0.05) by Tukey's HSD.

Table S8. Ovipositional inhibition effect of different oils against adults of *C. maculatus*.

	Percent OI (Mean±SD) of *C. maculatus* at different concentrations (µl/L) after 48 h					
Essential oils	10000	5000	2500	1250	625	Pooled mean
A. calamus	36.40±5.18	32.20±8.55	25.20±6.90	23.00±8.12	9.40±4.33	25.24±11.32[ab]
L. angustifolia	34.20±6.76	24.40±7.63	22.60±11.28	23.40±15.90	14.80±9.81	23.88±11.68[b]
H. spicatum	39.60±8.23	27.40±10.55	26.80±6.53	26.80±5.63	26.40±5.13	29.40±8.59[ab]
C. deodara	33.80±4.76	37.20±5.21	33.60±8.23	30.00±5.57	27.20±7.42	32.36±6.80[a]
P. wallichiana	29.80±9.15	15.60±8.96	28.20±8.41	31.80±4.38	13.20±9.39	23.72±10.92[b]
J. communis	45.60±6.11	36.20±9.68	25.60±6.80	37.00±32.70	20.00±11.27	32.88±17.72[a]
J. recurve	40.40±6.73	39.20±11.12	31.40±12.22	24.20±2.68	11.00±10.56	29.24±13.96[ab]
Pooled mean	37.11±7.91[a]	30.31±11.37[b]	27.63±8.81[b]	28.03±14.05[b]	17.42±10.37[c]	28.10±12.35
Oils	$F_{6,174}$=3.77; p<0.0001					
Concentrations	$F_{4,174}$=17.78; p<0.0001					
Oils × Concentrations	$F_{24,174}$=1.48; p>0.05					

Mean of five replications: Means followed by the same letter within a column are not statistically significant (p>0.05) by Tukey's HSD.

Table S9. Ovipositional inhibition effect of different oils against adults of *C. maculatus*.

	Percent OI (Mean±SD) of *C. maculatus* at different concentrations (µl/L) after 72 h					
Essential oils	10000	5000	2500	1250	625	Pooled mean
A. calamus	37.80±6.50	34.20±4.15	31.60±3.65	28.60±3.85	9.00±9.00	28.24±11.58[b]
L. angustifolia	35.00±7.11	24.80±7.89	22.80±10.01	18.00±5.74	14.60±6.84	23.04±9.97[b]
H. spicatum	39.00±6.44	28.20±8.76	25.20±6.22	24.20±5.45	25.00±4.00	28.32±8.09[b]
C. deodara	37.80±6.22	38.20±5.40	39.00±4.53	35.20±4.76	30.20±3.42	36.08±5.58[a]
P. wallichiana	27.60±9.55	17.00±8.92	28.00±7.51	31.20±5.07	16.µ80±4.82	24.12±9.16[b]
J. communis	41.00±6.00	28.60±16.36	29.40±7.67	24.80±10.30	13.80±7.98	27.52±12.97[b]
J. recurve	37.80±6.87	34.80±9.04	31.60±8.20	24.60±1.52	15.00±7.42	28.76±10.56[b]
Pooled mean	36.57±7.58[a]	29.40±10.83[b]	29.65±8.12[b]	26.65±7.41[b]	17.77±9.04[c]	28.01±10.53
Oils	$F_{6,174}$=8.35; p<0.0001					
Concentrations	$F_{4,174}$=30.58; p<0.0001					
Oils × Concentrations	$F_{24,174}$=2.20; p<0.0001					

Mean of five replications: Means followed by the same letter within a column are not statistically significant (p > 0.05) by Tukey's HSD.

Table S10. Ovipositional inhibition effect of different oils against adults of *C. chinensis*.

Percent OI (Mean±SD) of *C. chinensis* at different concentrations (µl/L) after 24 h						
Essential oils	10000	5000	2500	1250	625	Pooled mean
A. calamus	38.80±9.26	25.60±9.76	21.00±2.55	14.40±13.67	20.60±3.78	24.08±11.59^{b}
L. angustifolia	30.00±11.72	26.00±7.48	20.80±12.00	18.20±7.36	15.20±10.38	22.04±10.62^{b}
H. spicatum	26.00±11.11	26.80±12.01	23.00±10.86	21.80±9.98	19.20±5.80	23.76±9.90^{b}
C. deodara	38.40±6.50	34.30±5.32	29.80±6.38	19.60±9.07	17.60±5.50	27.96±10.31^{ab}
P. wallichiana	42.40±4.83	39.20±2.77	37.60±2.51	29.60±2.07	25.40±5.08	34.84±7.28^{a}
J. communis	46.40±6.27	32.40±4.22	27.00±7.21	19.80±5.36	18.00±8.89	28.72±12.05^{ab}
J. recurva	30.20±12.93	24.00±9.25	24.40±8.14	18.80±10.26	17.80±6.83	23.04±9.96^{b}
Pooled mean	36.03±11.03^{a}	30.06±8.83^{b}	26.23±9.07^{b}	20.31±9.23^{c}	19.11±6.99^{c}	26.35±10.99
Oils	$F_{6,174}$=7.45; p<0.0001					
Concentrations	$F_{4,174}$=25.18; p<0.0001					
Oils × Concentrations	$F_{24,174}$=0.96; p>0.05					

Mean of five replications: Means followed by the same letter within a column are not statistically significant (p > 0.05) by Tukey's HSD.

Table S11. Ovipositional inhibition effect of different oils against adults of *C. chinensis.*

	Percent OI (Mean±SD) of *C. chinensis* at different concentrations (µl/L) after 48 h					
Essential oils	10000	5000	2500	1250	625	Pooled mean
A. calamus	36.20±5.26	32.40±5.41	25.20±5.67	21.40±11.33	22.60±7.67	27.56±8.98[ab]
L. angustifolia	30.00±11.83	28.40±8.65	24.00±10.25	25.20±8.61	22.60±9.23	26.04±9.37[ab]
H. spicatum	28.80±8.29	29.60±12.30	25.60±5.28	25.80±10.35	23.40±7.83	26.64±8.69[ab]
C. deodara	38.60±6.02	33.60±5.98	29.20±4.87	19.20±8.41	16.60±6.07	27.44±10.34[ab]
P. wallichiana	40.20±6.72	39.00±1.87	35.20±3.27	26.20±5.44	19.60±7.92	32.04±9.52[a]
J. communis	31.80±5.89	30.00±4.00	22.00±3.24	18.80±4.87	13.40±2.41	23.20±8.03[b]
J. recurva	32.80±14.02	28.40±10.97	30.40±14.36	20.20±8.70	16.40±9.44	25.64±12.47[ab]
Pooled mean	34.05±9.02[a]	31.62±7.96[ab]	27.37±8.21[bc]	22.40±8.32[cd]	19.23±7.76[d]	26.94±9.87
Oils	$F_{6,174}$=2.73; p<0.05					
Concentrations	$F_{4,174}$=20.16; p<0.0001					
Oils × Concentrations	$F_{24,174}$=0.81; p>0.05					

Mean of five replications: Means followed by the same letter within a column are not statistically significant (p>0.05) by Tukey's HSD.

Table S12. Ovipositional inhibition effect of different oils against adults of *C. chinensis*.

	Percent OI (Mean±SD) of *C. chinensis* at different concentrations (µl/L) after 72 h					
Essential oils	10000	5000	2500	1250	625	Pooled mean
A. calamus	42.60±5.86	33.80±5.49	27.00±5.96	23.60±10.43	19.00±6.36	29.20±10.61^{ab}
L. angustifolia	31.40±9.58	30.20±6.87	28.40±9.53	26.80±3.96	22.80±13.03	27.92±8.86^{ab}
H. spicatum	34.80±6.46	32.80±10.57	31.60±6.65	30.00±7.71	26.00±4.47	31.04±7.44^{a}
C. deodara	40.20±3.49	34.20±4.27	30.20±3.96	19.80±7.85	16.20±2.50	28.12±10.10^{ab}
P. wallichiana	40.00±3.39	37.40±0.89	36.40±2.61	27.20±1.92	22.20±5.89	32.64±7.58^{a}
J. communis	40.40±4.56	28.80±7.15	25.60±7.33	22.20±2.59	19.40±3.78	27.28±8.93^{ab}
J. recurva	31.60±6.27	23.20±6.50	33.60±3.05	19.60±7.70	17.00±7.31	25.00±8.86^{b}
Pooled mean	37.28±6.45^{a}	31.48±7.36^{b}	30.40±6.54^{b}	24.17±7.14^{c}	20.37±7.11^{c}	28.74±9.12
Oils	$F_{6,174}$=3.77; p<0.0001					
Concentrations	$F_{4,174}$=36.46; p<0.0001					
Oils × Concentrations	$F_{24,174}$=1.58; p>0.05					

Mean of five replications: Means followed by the same letter within a column are not statistically significant (p > 0.05) by Tukey's HSD.
